# Data on the quality and methods of studies reporting healthcare costs of post-prostate biopsy sepsis

**DOI:** 10.1016/j.dib.2019.104307

**Published:** 2019-07-25

**Authors:** Mark N. Alshak, Michael D. Gross, Jonathan E. Shoag, Aaron A. Laviana, Michael A. Gorin, Art Sedrakyan, Jim C. Hu

**Affiliations:** a Weill Cornell Medical College, New York, NY, USA; b Department of Urology, Weill Cornell Medicine, New York, NY, USA; c Department of Urologic Surgery, Vanderbilt University Medical Center, USA; d The James Buchanan Brady Urological Institute and Department of Urology, Johns Hopkins University School of Medicine, Baltimore, MD, USA; e Department of Health Services and Policy Research, Weill Cornell Medicine, New York, NY, USA

**Keywords:** Sepsis, Health care costs, Prostate, Biopsy, Prostatic neoplasms, Cost and cost analysis, Urology

## Abstract

This data article presents the supplementary material for the review paper “Healthcare Costs of Post-Prostate Biopsy Sepsis” (Gross et al., 2019). A general overview is provided of 18 papers, including the details about year and journal of publication, country of dataset, data population characteristics, cost basis, and potential for bias evaluation. Quality assessment and the risk of bias of the 18 papers are detailed and summarized.

**Table:** Specifications Table

Subject	Medicine
Specific subject area	Urology
Type of data	Tables, figure
How data were acquired	Review and analysis of the relevant literature searched through Ovid MEDLINE, CINAHL (EBSCO), and Science Direct for datasets
Data format	Raw, analyzed
Parameters for data collection	18 articles overviewed and analyzed here were obtained through an extensive literature review where titles and abstracts of 874 articles were screened, 103 articles were reviewed in full against inclusion/exclusion criteria, and 18 datasets were found to meet the inclusion criteria.
Description of data collection	18 articles, identified as relevant through the above search and screening process were analyzed by extracting the relevant data such as author, year of publication, dataset location, aims of the dataset, outcome measures, and data.
Data source location	United States, Canada, New Zealand, France, Italy, Sweden, Australia, Netherlands, UK, Denmark
Data accessibility	All of the data is provided in this article.
Related research article	Gross, MD, Alshak MN, Shoag JE et al. Healthcare Costs of Post-Prostate Biopsy Sepsis. Urology. 2019. https://doi.org/10.1016/j.urology.2019.06.011

**Table:** 

**Value of the data** • The data serves as a way to provide greater insight into the various datasets examining the cost of post-prostate biopsy sepsis. • The data assists readers in understanding the review article [1] about the costs of post-prostate biopsy sepsis. • The data, along with the accompanying research article [1], provides an example of how to assess the quality and risk of bias of the included papers that can be used in other cost reviews. • The data provides greater detail in how sepsis and cost were derived in each dataset and how the risk of bias of each dataset was evaluated.

## 1 Data

The data in this article consists of additional and expanded tables and figures provided in a systematic review [1] of the literature including 18 research papers [2], [3], [4], [5], [6], [7], [8], [9], [10], [11], [12], [13], [14], [15], [16], [17], [18], [19], as well as the methodological and bias evaluation of these papers.

The data includes additional variables and characteristics included as a supplement to the systematic review [1]. Methodological and bias evaluation was conducted by two separate evaluators and are thoroughly described here in more detail.

**Fig. 1:**
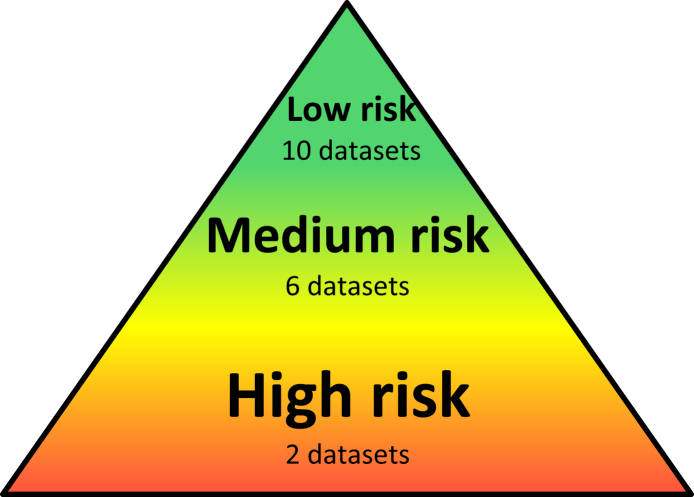
Number of datasets at low, medium, and high risk of bias.

Table 1 is an overview of the included datasets, comprised of author year, journal of publication, cases of sepsis and overall biopsies included in the dataset, the source of the data, and median age of men at biopsy. Table 2 is a breakdown of the datasets including country where data was gathered, publication year, and whether the dataset was a single institution, multi-institution, state-wide, or national dataset. Table 3 is a detailed description of how cost within their specific cohort was determined for each dataset. Table 4 is the assessment of bias using the Newcastle-Ottawa Quality Assessment Scale. Fig. 1 is the number of datasets at low, medium, and high risk of bias.

**Table 1:** Overview of the included datasets.

Author (year), Country	Journal	Cases of Sepsis (Overall biopsies)	Data source	Median Age
Evans et al. [2] (US)	Open Forum Infectious Diseases	5385 (515,045)	MarketScan Database (National database)	62, >40 years old
Nam et al. [3] (Canada)	The Journal of Urology	781 (75,190)	Canadian hospital and cancer registry administrative databases (National database)	b
Halpern et al. [4] (US)	The Journal of Urology	151 (9,893)	New York Statewide and Research Cooperation System (SPARCS) (State-wide database)	b
Bruyere et al. [5] (France)	The Journal of Urology	76 (2,718)	Groups throughout France. (Multi-institutional dataset)	b
Williamson et al.)[6] (New Zealand)	Clinical Infectious Diseases	47 (3,120)	Auckland City Hospital (Single institutional dataset)	61.4
Sanders et al. [7] (New Zealand)	ANZ Journal of Surgery	40 (1,421)	Public and private hospitals (Multi-institutional dataset)	66
Carignan et al. [8] (Canada)	European Urology	32 (5,798)	The Center Hospitalier Universitaire de Sherbrooke (Single institutional dataset)	66.7
Feliciano et al. [9] (US)	The Journal of Urology	19 (1,273)	Brooklyn and Manhattan campuses of New York Harbor VA Hospital (Multi-institutional dataset)	66.7
Pinkhasov *et al.*[10] (US)	BJU International	12 (1000)	Hershey Medical Center (Single institutional dataset)	63.8
Carmignani *et al.*[11] (Italy)	International Urology and Nephrology	9 (447)	Three centers (Multi-institutional dataset)	65
Adibi *et al.*[12] (US)	The Journal of Urology	11 (290)	University of Texas Southwestern Medical Center (Single institutional dataset)	b
Remynse *et al.*[13] (US)	Open Access Journal of Urology^c^	6 (197)	Urology Associates of Battle Creek (Single institutional dataset)	b
Duplessis et al. [14] (US)	Urology	3 (103)	Naval Medical Center San Diego (Single institutional dataset)	b
Larsson et al. [15] (Sweden)	Prostate Cancer and Prostatic Diseases	1 (298)	Huddinge University Hospital (Single institutional dataset)	64
Batura *et al.*[16] (UK)	Journal of Antimicrobial Chemotherapy	1813-2610^a^	England and Wales (National database)	b
Roth et al. [17] (Australia)	BJU International	218 (34,865)	Department of Health's Victorian Admitted Episodes Data Set (Multi-institutional dataset)	b
Chiu et al. [18] (The Netherlands)	BJU International	92 (10,747)	Rotterdam section of the European Randomized Study of Screening for Prostate Cancer (National database)	68
Thomsen et al. [19] (Denmark)	Scandinavian Journal of Urology	37 (317)	Rigshospitalet (Single institutional database)	65

a = estimated.

b = median age not reported.

c = Journal was renamed in 2013 to *Research and Reports in Urology*.

**Table 2:** Breakdown of the included datasets.

Country	Number of included datasets
United States	7
Canada	2
New Zealand	2
France	1
United Kingdom	1
Italy	1
Sweden	1
The Netherlands	1
Denmark	1
Australia	1
Publication year	Number of included datasets
2017	3
2015	3
2013	3
2012	5
2011	1
2010	1
2008	1
1999	1
Type of Dataset	Number of included datasets
Single institutional dataset	8
Multi-institutional dataset	5
National database	4
State-wide database	1

**Table 3:** Detailed descriptions of cost determination of included datasets.

Author	Average cost of urosepsis ($)	CPI IP Adjusted Cost ($)	Means of cost determination
Evans et al. (2017)	14,499	19,121	Total gross payments to all providers who submitted claims for covered services, including total gross payments to the hospital
Halpern et al. (2017)	4,219	5,076	Total charges as documented in SPARCS database
Adibi*et al.* (2013)	5,900	8,959	Average cost of hospitalization from sepsis in this specific hospital
Remynse *et al.* (2011)	5,410	8,215	Average hospital reimbursement from insurance in this specific hospital
Duplessis et al. (2012)	5,711	8,672	Average cost of hospitalization from sepsis in this specific hospital
Larsson et al. (1999)	849	2,720	Cost of hospital expenditures from this specific case of sepsis
Batura *et al.* (2013)	6,944^a^	8,801^a^	Average bed cost (estimate provided by finance department, North West London Hospitals) multiplied by average length of stay of sepsis
Roth et al. (2015)	6,844	9,026	Data provided by the Department of Health and Human Services from a payer's perspective
Chiu et al. (2017)	3,102	3,578	Average daily cost of hospital admission for post-biopsy complication multiplied by median length of stay
Thomsen et al. (2015)	3,416	4,329	Average cost at this specific institution for 10 randomly selected patients with admission following biopsy

Table adapted from Table 1 of Healthcare Costs of Post-Prostate Biopsy Sepsis.^1^

a = estimated.

**Table 4:** Assessment of bias using the Newcastle-Ottawa Quality Assessment Scale.

First Author (Year)	Selection	Exposure		Outcome		
	Representativeness of the sample	Ascertainment of exposure	Assessment of outcome	Same method of assessment for entire sample	Adequacy of follow up	Total
Evans (2017)	1	1	0	1	1	4
Nam (2010)	1	1	0	1	1	4
Halpern (2017)	1	1	0	1	1	4
Bruyere (2015)	1	1	1	0	1	4
Williamson (2012)	0	1	0	1	1	3
Sanders (2013)	0	1	0	1	1	3
Carignan (2012)	0	0	0	1	1	2
Feliciano (2008)	1	1	0	1	1	4
Pinkhasov (2012)	1	1	0	1	1	4
Carmignani (2012)	1	1	1	1	1	5
Batura (2013)	1	1	0	0	0	2
Roth (2015)	1	1	1	1	0	4
Chiu (2017)	1	1	1	1	1	5
Thomsen (2015)	0	1	0	1	1	3
Adibi (2013)	1	1	0	1	0	3
Remynse (2011)	0	1	0	1	1	3
Duplessis (2012)	1	1	1	1	0	4
Larsson (1999)	0	1	1	1	0	3

A score of 0–2 indicates high risk of bias, 3 is moderate risk of bias, 4–5 is low risk of bias.

Table referenced from supplementary Table 1 of Healthcare Costs of Post-Prostate Biopsy Sepsis.^1^

## 2 Experimental design, materials, and methods

The review was performed following the instructions set forth by the Preferred Reporting Items for Systematic Reviews and Meta-Analyses (PRISMA) statement [20]. All 874 articles were reviewed at the abstract level. Articles that were reviewed in full included abstracts that commented on post-prostate biopsy sepsis cost. Articles that were reviewed in full were systematically evaluated by two reviewers (MG and MA) to assess for eligibility of inclusion/exclusion criteria. Articles were included if they had individual or system-wide cost or burden of post-prostate biopsy hospital admission. Extraction of data then took place. A modified STROBE criterion was used to evaluate dataset quality metrics [21]. Healthcare costs of hospitalization for infection following prostate biopsy was defined as the primary outcome.

Standard aspects of reviewing and extracting data, as described by the Preferred Reporting Items for Systematic Reviews and Meta-Analyses (PRISMA) statement [20], were adopted for this review. The extracted aspects included the following:

1. General information about the included paper: [authors, year of publication, journal of publication, location of dataset]
2. Dataset characteristics: [primary and secondary outcomes, data design, and overview of the methods]
3. Data population: [number of included patients, inclusion/exclusion of patients, primary indication for biopsy, demographics]
4. Exposure, outcomes, and cost: [details of the exposure, detailed primary and secondary outcome analysis, how the primary and secondary outcomes were obtained, cost of primary outcome (sepsis)]
5. Data: [data of included papers]

General information, dataset characteristics, selected data populations, source of data, exposure with primary outcome of sepsis, and data extracted from the 18 selected papers are outlined in Table 1. In the table we describe authors, year of publication, country of dataset, journal of publication, overall population examined, number of sepsis cases reported, specifics of data location and design, and selected demographics when provided in the respective papers. Table 2 includes counts of the countries of dataset, years of publication, and methodology performed by each paper.

Healthcare costs were analyzed for 10 datasets and directly and indirectly evaluated in the context of expenditures for an episode of sepsis related to prostate biopsy. Each dataset had its own method of determining costs, which is described in Table 3, along with author, year of publication, average cost of sepsis, and the inflation-adjusted cost [22]. All costs were adjusted for inflation to the May 2018 urban and inpatient hospital service consumer price indices [22]. This method is modeled after the same approach used by the Agency for Healthcare Research and Quality [23]. If a paper did not specify what dollar year their costs were originally derived from, the year of publication was used. For the international cohorts, all currency amounts were compared to the U.S. dollar using the historical exchange rate from the federal reserve before adjusting for inflation as described above [24].

We then assessed the quality of each paper according to the Newcastle-Ottawa Quality Assessment Scale for cohort analysis [25]. This was considered the most appropriate evaluation of bias as it:

1. Is widely used and accepted for quality assessment
2. Demonstrates both inter- and intra-rater reliability
3. Demonstrates criterion and construct validity
4. Demonstrates objectivity, as questions are well defined and easy to understand
5. Is described as a tool that evaluates papers that are included in this type of analysis

Five questions were used from the Newcastle-Ottawa Quality Assessment Scale, given the observational nature of the included papers and lack of control groups. Questions were evaluated by two independent reviewers, with conflicts being resolved with further analysis of the papers. Questions are answered with a yes (1) when information was directly available in the text of the papers or no (0) when information in the text was either directly contradictory to the question, not sufficient or specific enough to answer properly, or not available to analyze. Detailed analysis of the risk of bias assessment, along with more detail of answers to individual questions, are reported in Table 4 [1].

For the selection category, representativeness of the exposed cohort was used to evaluate bias. A yes (1) in this category means that the exposed cohort either (a) truly or (b) somewhat represented the average man of average age undergoing prostate biopsy with no increased risk of sepsis or hospital admission due to other co-morbidities in the community. For our analysis, we defined truly or somewhat representative of the average man if patients were all chosen within a specified timeframe in multiple institutions. A no (0) means that the selected group of users were from a specific cohort (i.e. nurses, volunteers, all from a single institution) or there was no description of the derivation of the cohort.

For the exposure category, ascertainment of exposure was used to evaluate bias. A yes (1) in this category means that the exposed group was found by either (a) secure records (e.g. surgical records, national database, billing codes) or (b) structured interview (e.g. medical records). A no (0) in this category means that the exposed was ascertained by either (a) a written self-report from the patient or (b) there was no description of how patients who had a prostate biopsy was chosen.

For the outcome category, multiple questions were used to assess bias. The first question includes the assessment of outcome. A yes (1) for this question means that the outcome was assessed by either (a) independent blind assessment (i.e. an assessment from a physician) or (b) record linkage (i.e. medical records, billing codes, databases). A no (0) for this question means that the outcome in questions was either self-reported or had no description of how the outcomes were chosen. The second question is whether the exposure and outcome had the same assessment or if they differed. A yes (1) means that the exposure and outcome had the same means of assessment, where a no (0) means that the way that each was selected differed. The last question in this category is whether there was adequate follow-up of patients. A yes (1) means that follow-up was complete for all subjects with an adequate amount of time given for follow-up, here defined as at least 1-month post-biopsy. A no (0) constitutes that follow-up was not complete for all subjects, patients were lost to follow-up, patients were evaluated for outcomes before 1-month and not evaluated again, or there was no comment on how follow-up was defined.

After completing the risk of bias of each dataset, we found that among 18 datasets included, 10 were of low risk, 6 were of medium risk, and 2 were of high risk (Fig. 1).
